# A Feminine Care Clinical Research Program Transforms Women’s Lives

**DOI:** 10.5539/gjhs.v7n4p45

**Published:** 2014-12-16

**Authors:** Ghebre E. Tzeghai, Funmilayo O. Ajayi, Kenneth W. Miller, Frank Imbescheid, Jack D. Sobel, Miranda A. Farage

**Affiliations:** 1 The Procter and Gamble Company, Cincinnati, Ohio, USA; 2 The Procter & Gamble Company, Geneva, Switzerland; 3 Wayne State University School of Medicine, and Harper Hospital, Detroit, Michigan, USA

**Keywords:** clinical, BTK, Behind the Knee, Quality of Life, FQoL, Feminine Hygiene, Feminine Care, Feminine wellness, safety, women’s health

## Abstract

Feminine hygiene products and menstruation education have transformed the lives of women throughout the world. The P&G Feminine Care Clinical Innovation Research Program has played a key role by expanding scientific knowledge as well as developing technical insights and tools for the development of feminine hygiene products. The aim has been to meet the needs of women throughout their life stages, advancing their urogenital health beyond just menstruation, as well as helping to understand the role of sex hormones in various important health issues that women face. This review article highlights key contributions and research findings in female hygiene products, urogenital health research, and method development. The clinical research team focused on utilizing the results of clinical safety studies to advance the acceptance of feminine hygiene products world-wide. Key findings include that perception of skin sensitivity is not limited to the facial area, but is also relevant to the body and the genital area. Also, they shed light on the role of estrogen in autoimmune diseases as well as premenstrual syndrome. Efforts in the method development area focused on innovative tools that are reliable, predictive of clinical trial results and capable of measuring wear comfort, genital skin health, and the impact of product use on the consumer’s quality of life. A novel method, behind-the-knee (BTK) test, developed to model irritation under normal wear conditions, was the first to account for both chemical and mechanical sources of irritation. The method has been accepted by the FDA as a substitute in clinical trials in some cases, and by American Society for Testing and Materials as a global standard test method. Additional proprietary methods were developed to enhance visual grading of irritation using cross-polarized light, to measure the amount of lotion transferred from sanitary pads, and to evaluate the skin mildness. Finally, the Farage Quality of Life tool was created to measure consumer’s well-being. Based on the results of this extensive clinical research and the newly developed testing methods, the changing needs of women throughout their life stages are better met.

## 1. Introduction

Modern feminine hygiene products and menstruation education have transformed the lives of women throughout the world. Innovative research has played a key role in global efforts by developing technical insights and tools for the development of next generation feminine hygiene products aimed at meeting the needs of women throughout their life stages.

Cultural acceptance and the use of disposable pads and tampons has taken considerable time. Almost two decades passed after the introduction of commercial sanitary pads before they supplanted the use of cloth in Western industrialized countries (Friedman, 1981). Even today, tampons are not widely accepted in the developing world because of cultural beliefs and taboos about menstrual blood and products that inhibit the natural flow of menstrual blood out of the body (Snow, 1983). Clinical safety testing, feminine hygiene and puberty education, and the changing roles of women in society have driven the acceptance and the desire for even better feminine hygiene products to meet the changing needs of women as they advance in age.

Education has been a critical factor in driving acceptance of feminine hygiene products and improving the lives of women around the world. Manufacturers of feminine hygiene products have taken a leading role in these efforts. One corporate partnership with several organizations supported the delivery of education and product sampling programs in New Zealand and Australia, reaching 200,000 young women in 2012 (Kimberly-Clark Corporation, 2013). A similar effort in South Africa provided 12 000 young women with sanitary pads on Mandela Day (Kimberly-Clark Corporation, 2013). The first Always^®^/Whisper^®^ puberty-education program was initiated in North America in 1984. Since then, puberty education programs have expanded to reach young women in more than 65 countries with 500,000 education professionals using those manufacturer-provided puberty-education materials. Over the years, programs have evolved to include not only school-based programs but also extra-curricular programs and self-directed learning programs. Examples of self-directed programs include websites (e.g., www.beinggirl.com), YouTube peer-to-peer tutorials, a mobile phone app, and a Facebook community. Since 2006, Procter & Gamble (P&G) has partnered with numerous non-government agencies and organizations to bring school-based and community-based feminine hygiene and puberty education programs to young women around the world. These programs reach between 17 and 20 million young women annually (P&G Corporate Newsroom, 2012) including over 80% of fifth-grade girls in the US (Procter & Gamble, n.d.) as well as girls in Europe, Asia, Kenya, Tanzania, Uganda, Nigeria, Ghana, Nepal, Ethiopia, and South Africa. Recently P&G has committed to providing an additional 200 million puberty lessons between 2013 and 2016 in partnership with the United Nations Educational Scientific and Cultural Organization (UNESCO) (P&G Corporate Newsroom, 2012).

The transformational impact that feminine hygiene products have on the lives of women is difficult to appreciate in Western society after decades of acceptance and use. The impact is better illustrated by improvements in the quality of life (QoL) (Farage, Rodenberg, & Chen, 2013) and future opportunities these products can provide in the developing world, where girls can miss 10% to 20% of schools days after the onset of puberty (P&G Corporate Newsroom, 2007). A report in 2007, noted that education of girls in Africa and Asia continues to lag far behind that of boys (Ten, 2007) and that failure to address the relationship between menstrual hygiene and the drop-out rate of girls could jeopardize the achievement of several Millennium Development Goals set by the United Nations. Lack of reliable menstrual protection has been identified as one of the key reasons girls miss or drop out of school (Ten, 2007).

A recent study showed that puberty education along with access to reliable feminine protection can significantly improve school attendance and the quality of life for young women in Ghana (Murray, 2010; Scott, Dopson, Montgomery, Dolan, & Ryus, 2009). The study followed 183 menstruating girls from four villages in Ghana over a 5- to 6- month period after receiving puberty education and a supply of sanitary pads. On average, the rate of absenteeism was reduced significantly by slightly more than half from about 21% to 9% of school days. The vast majority (98.4%) of participants reported that they were better able to concentrate in school as a result of using sanitary pads. Negative experiences related to soiling and embarrassment declined, and measures of well-being improved. Participants also reported a strong preference for the pads over traditional methods, primarily due better protection against accidental soiling and reduced worry about embarrassing odors.

**Figure 1:**
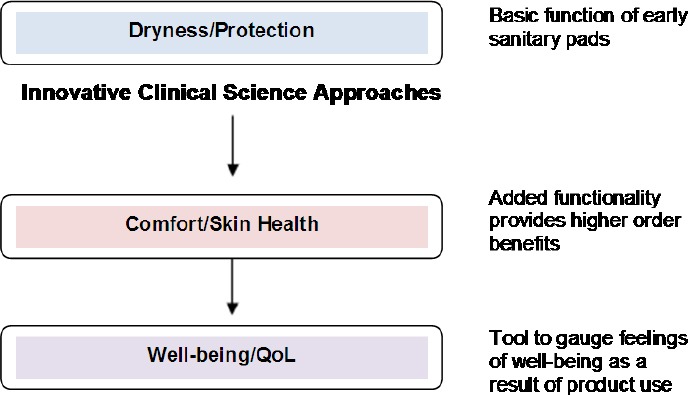
Evolution of Benefits Provided by P&G Feminine Hygiene Products

Our Feminine Care clinical innovation research program at P &G has contributed to the development of improved products through scientific insights and methods for the development of next generation products and future products. In addition to designing and conducting controlled clinical trials to establish the clinical safety of new products, researchers conduct basic urogenital research, publish their findings, collaborate with leaders in the field to publish reference books, and develop innovative methods and tools that are capable of quickly and reliably measuring desired product benefits that are predictive of clinical trial results. That program and its leadership have been recognized as a key player in increasing awareness of the health concerns of women’s genital areas and in ending the taboo over menstruation. The development of feminine hygiene products that go beyond providing only dryness and protection benefits to also providing additional consumer benefits such as comfort, genital skin health and overall feelings of well-being (Figure 1). Our research program has provided the advancement of scientific knowledge and understanding in the urogenital area which plays a role in developing and improving feminine hygiene products. This review article highlights the findings and contributions of this program related to sanitary pads in three areas: clinical science research, urogenital health research and methods development.

## 2. Clinical Research: Developing and Sharing Learnings From Clinical Trials

Feminine hygiene products are regulated by the Food and Drug Administration (FDA) in the US as medical devices. Product upgrades must be accompanied by technical data demonstrating that the new product is safe (non-irritating) and effective (protects from leakage) compared to a currently marketed product under practical use conditions (Farage, 2006a). Comparative clinical trials are considered the definitive way to demonstrate product safety and effectiveness. In these trials, a new product is compared to a marketed product among menstruating women over a 3-month period. Medical professionals conduct assessments of the genital area prior to and after the product is used for a period of time for any negative changes attributable to the product such as erythema. In addition, participants are asked to report any negative experiences during product use and provide feedback on their overall product experience at the end of the study.

Clinical trials on sanitary pads catalog findings for future reference and claims, as well as advance the understanding of the clinical safety and effectiveness for development of improved feminine hygiene products. A recently published review included clinical trials of products marketed in North America, Europe and Asia involving more than 1600 adolescent and adult subjects in two continents (Farage, Stadler, Elsner & Maibach, 2004). The results consistently showed that modern feminine hygiene products are effective and are not associated with clinically significant adverse dermatological, gynecological, or microbiological outcome compared to conventional pads (Farage, Bramante, Otaka, & Sobel, 2007; Farage, Elsner, & Maibach, 2007; Farage, Stadler, Elsner, & Maibach, 2004; Stadler, Tischler, Wambebe, Osisanya, & Farage, 2006).

The impact of global variations in pad usage, skin type, and ambient conditions on skin mildness of modern sanitary pads was evaluated by reviewing all relevant clinical trials in the literature and publishing a review of the findings (Farage, Elsner, & Maibach, 2007; Farage, Stadler, Elsner, & Maibach, 2004). The global review (Farage, Stadler, Elsner, & Maibach, 2004) included eight prospective clinical trials of sanitary pads conducted in the US, Mexico, Western Europe, Eastern Europe and Africa. It was hypothesized that 1) higher daily usage of pads in regions such as Japan compared to Nigeria could influence skin compatibility, 2) dark skin might be less susceptible to certain irritants than fair skin, and 3) regions with high temperature and humidity might show differences in skin compatibility relative to regions with cold, dry conditions. Despite this diverse range of conditions, modern pads showed no significant adverse effects on the skin compared to traditional pad designs. Furthermore, study participants generally preferred modern pads for performance and comfort.

Several clinical trials conducted in developing countries to provide additional assurance about the clinical safety of modern sanitary pads. For example results of a clinical trial conducted among 289 healthy Nigerian women showed comparable low levels of irritation for both pads tested, (Always Ultra and a local competitor pad); however, the Always pad was preferred for comfort, protection from soiling, and fit to underwear (Stadler, Tischler, Wambebe, Osisanya, & Farage, 2006).

It was speculated in the literature that the use of panty liners could promote vulvovaginal candidiasis (VVC) and urinary tract infections (UTIs). Scientific evidence including a series of 13 randomized prospective trials of panty liners or ultra-thin pads demonstrated no clinically significant adverse effects either on the skin or the genital microflora. It was concluded that panty liners are safe when used as intended and do not promote VVC or UTI (Farage, Bramante, Otaka, & Sobel, 2007; Stadler, Tischler, Wambebe, Osisanya, & Farage, 2006).

## 3. Advancing Urogenital Health Understanding and Science

### 3.1 Urogenital Health Research

The Fem Care urogenital research program was established to address the lack of scientific understanding and ongoing research in the area of urogenital science. The objective was to address knowledge gaps critical to current and future product initiatives. A secondary objective was to encourage additional interest and research in the area by broadly sharing research results and collaborating with external experts. To address the knowledge gaps, over 230 peer-reviewed articles have been published and findings have been shared at dozens of international professional meetings. Learnings from this work have been used for developing training for medical professionals and educational materials used by non-profit organizations. In addition, four reference books have been published in collaboration with experts in the area.(Farage & Maibach, 2006; Farage, Miller, & Maibach, 2010b; Surber, Elsner, & Farage, 2011; Farage, Miller, Woods, & Maibach, 2015;)

An early focus of the research was to develop an understanding of how the tissue structure and physiology of the vulva differs from cutaneous skin in order to adapt available dermatological test methods to the measurement of irritation in the genital area. (Farage & Maibach, 2004; Farage, Bjerke, Mahony, Blackburn, & Gerberick, 2003) Typically, skin irritation is assessed visually by expert graders and measured using a scoring scale of 0 to 4. It was not known whether irritation scores noted by expert graders correlated with panelists’ perception of irritation. Studies showed a correlation between an increased irritation score and a higher percentage of panelists reporting adverse sensory effects (Farage, Santana, & Henley, 2005).

Currently marketed feminine hygiene products are generally non-irritating. As a result, there is a need for more sensitive approaches to enhance the ability to measure differences in irritation potential between mild products. Several approaches have been explored including testing products among panelists with sensitive skin, measurement of early signs of irritation, and the use of enhanced visual grading using cross polarized light (Farage, 2008c).

Enhanced visual grading was used to evaluate patients previously diagnosed with vulvodynia (previously known as vulvar vestibulitis syndrome) on the basis of sensory testing alone (Farage, Singh, & Ledger, 2009). Findings showed that despite the normal appearance of the labia in these patients, examinations using cross polarized light (V600 Visualization System, Syris Scientific LCC, Gray, Maine) revealed subsurface inflammation. This instrumental approach represents a promising new means of objectively diagnosing subclinical inflammation in disorders that have been characterized solely by sensory symptoms (Farage, Miller, Gerberick, Ryan, & Maibach, 2010; Farage, Singh, & Ledger, 2009).

Measurement of cytokines, mediators of the inflammatory process, was explored as an early measure of skin irritation. Compared to normal skin, skin treated with sodium lauryl sulfate (SLS) to induce minimal visible irritation was observed to have significantly higher level of cytokines IL-1α and a significantly lower ratio of IL-1RA/IL-1α (Farage, 2008c).

Several avenues have been explored in the area of sensitive skin. Evaluations were conducted to determine whether women with pre-existing vulvar erythema represent a more sensitive skin population. Results showed a statistical correlation between the presence of vulvar erythema and the development of facial redness after repeated application of topical products (Farage, Bowtell, & Katsarou, 2006). A questionnaire-based algorithm was developed and validated to identify panelists predisposed to having skin issues (Farage, Bowtell, & Katsarou, 2010). A global survey was conducted to understand the prevalence of sensitive skin. Results showed differences in self-reported sensitive skin across geographies with the highest reports in Asia (89.8%), followed by Europe (87.7%), Latin America (85.4%) and the US (76.8%). Overall, 68% of women claimed they have sensitive skin and 56% specified that they have sensitive genital skin (Farage, 2008d; Farage, Miller, Wippel, Berardesca, Misery, & Maibach, 2013). These studies were the first to establish that perception of skin sensitivity is not limited to the facial area, but may also involve the body and the genital area.

The skin’s response to irritants is also known to change with age. Visible signs of erythema are delayed and less intense in older people than in younger people. Signs of skin irritation in older people are more likely to be in the form of chronic dryness and itch (Farage, 2008c). In a comprehensive evaluation of skin irritation, a consistent trend was observed toward decreased erythema and increased irritation with age (Farage, Miller, Gerberick, Ryan, & Maibach, 2010). In recent clinical testing, it was observed that significantly more postmenopausal women preferred using wet wipes containing proprietary lotion ingredients compared to dry toilet tissue for their intimate feminine hygiene needs compared to pre-menopausal women (Farage, Stadler, Chassard, & Pelisse, 2008). It was hypothesized that this subgroup preferred using wet wipes due to skin atrophy and dryness that accompany estrogen depletion. These findings suggest that opportunities exist to develop products that are tailored to the genital skin condition and preferences of younger and older women.

### 3.2 Advancement of Scientific Understanding

Over recent years, attention has been given to the role of estrogen in immune processes and aging in women (Ackerman, 2006; Ansar Ahmed, Penhale, & Talal, 1985; Olsen & Kovacs, 2001). High estrogen levels stimulate antibody production, resulting in higher levels of circulating interleukin-1 (IL-1), IL-4, and interferon-gamma (IFN-γ) and higher graft rejection rates in females as compared to males (Ackerman, 2006; Ansar Ahmed, Penhale, & Talal, 1985). Estrogen is implicated as the main effector in the significant gender discrepancy that exists in many autoimmune diseases (Olsen & Kovacs, 2001). For example, the incidence of systemic lupus erythematosus (SLE) in women, for example, is estimated to be twenty times as high as that in men (Selmi, Brunetta, Raimondo, & Meroni, 2012). Also, the severity of SLE dermatitis was shown to fluctuate in concert with estrogen levels. It is known that intensity of patch-test reactions varies over the menstrual cycle, with skin reactivity to both irritants and antigenic substances increasing during the premenstrual phase (Farage, Berardesca, & Maibach, 2009b, 2010). The strong association between premenstrual syndrome (PMS) and cutaneous diseases, which are known to be of autoimmune origin, suggests a possible role of autoimmunity for PMS—a disease whose etiology is still a profound mystery (Farage, Miller, Ajayi, & Ledger, 2010). For millions of women across the globe who suffer from autoimmune diseases (including cutaneous autoimmune disorders) and PMS, the ultimate research goal would be an understanding of the extremely complex interactions between the neurological, endocrine, and immune systems in order to define therapies with the potential to abort disease pathogenesis.

## 4. Innovative Methods: Measuring Skin Health Benefits and Product Impact on Well-Being

Recent product innovations have utilized new topsheet materials that provide a more cloth-like feel (Farage et al., 2005; Farage, Stadler, Elsner, Creatsas, & Maibach, 2005) and topsheets impregnated with lotions and emollients for skin health benefits (Farage & Warren, 2011). Emollients, both natural and synthetic, are designed to soften and soothe scaly and dry skin. This is generally accomplished by lubricating, moisturizing and/or occluding the skin. Facial tissues and diapers containing lotions and emollients have been widely accepted by consumers for their skin health benefits. Facial tissues with lotion impregnated into the surface have been shown to soothe irritated skin around the nose for people with the common cold or allergies (Farage, Ebrahimpour, Steimle, Englehart, & Smith, 2007).

Conceptually, sanitary pads with topsheets containing lotions and emollients would be more comfortable to wear and provide genital skin health benefits, which would be accepted and appreciated by the consumer (Farage & Warren, 2011). From the development perspective, parameters to be defined would include an optimized lotion/emollient system for maximum skin health benefits and the mode of impregnation which would result in effective lotion transfer to the skin under normal wear conditions.

Clinical trials are time consuming (4-6 weeks) and costly (up to $250,000 per study). As such, they are not practical for routine testing of product prototypes. Instead, efforts were focused on developing innovative clinical methods and tools that quickly and reliably measure desired product benefits and predict in-use clinical trial results (Farage, 2008e; Farage, Miller, Gerberick, Ryan, & Maibach, 2010). Several of the clinical methods discussed below have been patented. The success of these efforts paved the way for development of a new generation of products that go beyond meeting basic consumer needs of dryness and protection to provide higher order benefits such as comfort, genital skin health and overall feelings of well-being (Figure 1).

### 4.1 Behind-the-Knee Method

**Figure 2:**
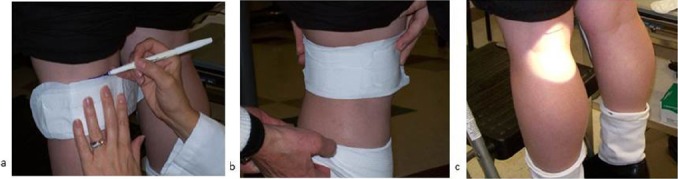
Behind-the-Knee Test Method

A novel method known as the Behind-the-Knee (BTK) test was developed to model irritation that can arise during normal wear conditions. This method takes into account both chemical and mechanical sources of irritation. (Farage, 2000b; Farage, Gilpin, Enane, & Baldwin, 2001; Farage, Meyer, & Walter, 2004; Farage, 2006b, 2008b, 2014). Mechanical sources of irritation are captured by applying the product to the popliteal fossa (behind the knee). To our knowledge, this was the first method developed to evaluate the frictional component of potential irritation from feminine hygiene and textile/fabric products. Turnaround time and costs are significantly reduced relative to in-use clinical trials such that routine evaluations of materials and prototypes are viable. In addition, two products can be tested simultaneously to provide a side-by-side comparison (Figure 2).

Test materials (pad, panty liner, topsheet, uncompressed tampon, fabric, facial tissues, etc.) are placed horizontally behind the knees (a) and held in place using an elastic athletic band of the appropriate size (b). After the 6 hr wear time, the band and test material are removed, the area is illuminated and visually scored for erythema or dryness by an expert grader (c).

**Figure 3:**
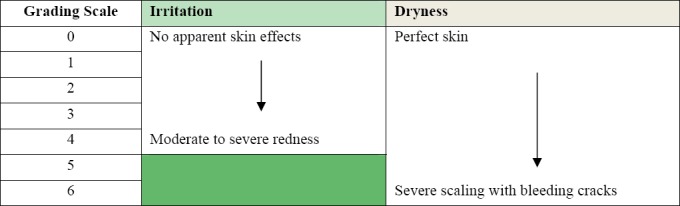
Grading Scales for Behind-the-Knee Method

In the BTK method, test materials (pads, panty liners, topsheets, uncompressed tampons, fabric) are applied to the back of each knee using an elastic athletic band and worn for 6 hr/day for 4-5 days. The test sites are graded daily by trained experts using grading scales for irritation and dryness (Figure 3). The athletic band provides close contact between the product and the skin. The pressure exerted by the athletic band has been found to be similar to real product wear conditions (Farage, Tucker, & Wang, 2012).

The BTK method has been validated by assessing its reproducibility (Farage, 2006b; Farage, 2008b) and its ability to detect differences between products, which were also found to be consistent with results of clinical trials (Farage, 2008b; Farage, Miller, & Ledger, 2011, 2013). The method has been accepted by FDA as substitute for in-use clinical trials, under certain conditions, for new sanitary pads and tampons with only minor product changes. It has also been approved by American Society for Testing and Materials (ASTM) as a global standard test method entitled: Protocol F2808-10 Standard Test Method for Performing Behind-the-Knee (BTK) Test for Materials that Come into Repeated or Extended Contact with the Skin.

#### 4.1.1 BTK-Lotion and Ingredient Transfer Method

The BTK method was adapted to provide an effective means of measuring differences in the amount of lotion and dye transferred from products to the back of the knee. This is known as the BTK-Lotion transfer method or BTK-ingredient transfer clinical method. Collection tape strips are applied to the back of the knee area prior to placement of the product. Collection tape strips are removed and the amount of emollient transferred is determined (Farage, 2010a; Farage & Warren, 2011). This propriety approach has been validated by comparing results from the BTK-Lotion transfer method with those obtained from in-use clinical testing (Farage, 2008a; Farage, 2010a).

#### 4.1.2 Effects of Various Lotion Concentrations on Skin Dryness in the BTK

**Figure 4:**
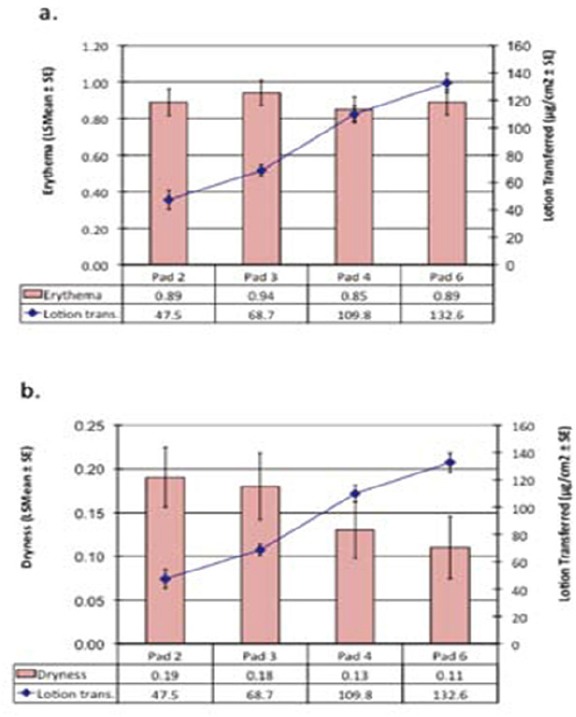
Effect of Lotion on Erythema and Skin Dryness

BTK studies were conducted on products identical in all respects except for the amount of lotion on the surface (pads 2, 3, 4 and 6). Reactions were scored visually prior to any treatment (baseline) and each day after removal of the samples. Figure 4 shows the overall mean scores (average of scores for days 1-5) for erythema (a) and dryness (b) for each product. The amount of lotion transferred for each test product is also indicated (Farage, Wang, Miller, Berardesca, Wilhelm, & Maibach, 2012). Results (Figure 4a) showed no significant differences in erythema scores as the amount of lotion transferred increases. However, skin dryness scores (Figure 4b) decreased with increasing level of lotion transferred. Paired comparisons for dryness scores showed significant differences between pads 2 and 4 (p=0.04), pads 2 and 6 (p=0.05) and pads 3 and 6 (p=0.05). These results illustrate the BTK showed the skin benefits of the lotion.

BTK studies were conducted on products identical in all respects except for the amount of lotion on the surface. Reactions were scored visually prior to any treatment (baseline) and each day after removal of the samples. The graph shows the overall mean scores (average of scores for days 1-5) for erythema (a) and dryness (b) for each product. The amount of lotion transferred for each test product is also shown.

- All values for treatment groups showed mean erythema and dryness scores significantly different from baseline values (p<0.0005 for all except Pad 6 dryness where p=0.0019).
- Paired comparisons for erythema; none were significantly different.
- Paired comparisons for dryness: Pad 2 and Pad 4, p=0.04; Pad 2 and Pad 6, p=0.05; Pad 3 and Pad 6, p=0.05. No other paired comparisons were significantly different.

(Courtesy of Household and Personal Care Today journal, Volume 1, 62-68, 2012)

### 4.2 Modified Forearm Controlled Application Test (mFCAT)

The Modified Forearm Controlled Application Test (mFCAT) was adapted to evaluate menstrual pads containing lotions (Farage, Wang, Miller, Berardesca, Wilhelm, & Maibach, 2012; Farage & Wilhelm, 2012). This patented clinical method was originally developed to evaluate the skin mildness of disposable baby wipes products (Farage, 2000a) and has been used to monitor the skin benefits of various lotion formulations on facial tissues (Farage, Ebrahimpour, Steimle, Englehart, & Smith, 2007). The mFCAT testing is conducted on the volar or inner surface of the forearms of panelists after the test sites are pretreated with 24 hour patches of sodium lauryl sulfate (SLS) to induce mild or moderate skin irritation. This is followed by repeated swiping of the test sites with the lotion-coated pads using a back and forth motion with sufficient pressure to ensure direct contact with the skin. Test sites are graded visually for irritation and dryness and/or evaluated using instrumental techniques to measure skin friction and skin barrier function.

#### 4.2.1 Effects of Different Lotion Amounts on Skin Barrier Effects

**Figure 5:**
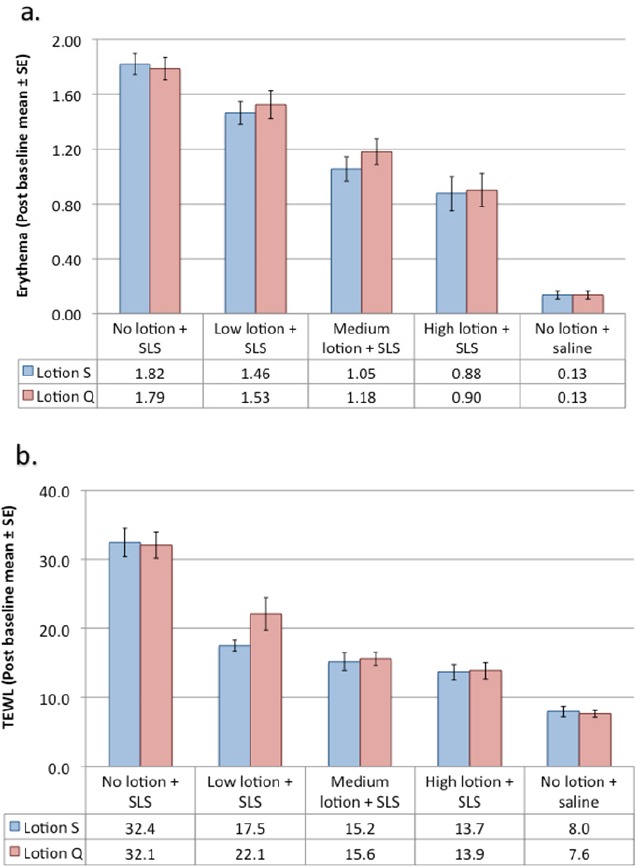
Effect on lotion concentration on skin barrier effects

Arm patch studies were conducted on two lotion formulations, Q and S (Farage, Wang, Miller, Berardesca, Wilhelm & Maibach, 2012). Test sites were pretreated with three different concentrations of lotion (high=825 µg/cm^2^, medium=495 µg/cm^2^, and low= 165 µg/cm^2^), then patched with 0.3mL of 0.5% SLS. Control sites received no pretreatment. The positive control was patched with SLS. The negative control was patched with saline. Sites were evaluated for erythema (a) and transepidermal water loss (TEWL) (b). The average of all scores after the baseline treatment is plotted in Figures 5a & b. Mean scores for erythema were significantly lower (p≤0.05) for both lotions at all dosing levels compared to the positive control (no lotion). The change in TEWL means for both lotions at all dosing levels significantly lower (p≤0.05) than for the positive control (p≤0.05). These results illustrate the protective effects of lotions tested.

Arm patch studies were conducted on the lotion formulations Q and S. Test sites were pretreated with three different concentrations of lotion (high=825 µg/cm^2^, medium=495 µg/cm^2^, and low= 165 µg/cm2), then patched with 0.3 mL of 0.5% SLS. Control sites received no pretreatment. The positive control was patched with SLS. The negative control was patched with saline. Sites were evaluated for erythema (a) and TEWL (b). The average of all scores post baseline are plotted.

(a) Erythema: Means for all treatments with lotions S and Q (high, medium and low) were significantly lower than the mean for the positive control (p≤0.05).
(b) Change in TEWL: The change in means for all treatments with lotions S and Q (high, medium and low) were significantly lower than for the positive control (p≤0.05).

(Courtesy of Household and Personal Care Today journal, Volume 1, 62-68, 2012).

#### 4.2.2 Effects of Topsheet on Skin Friction

**Figure 6:**
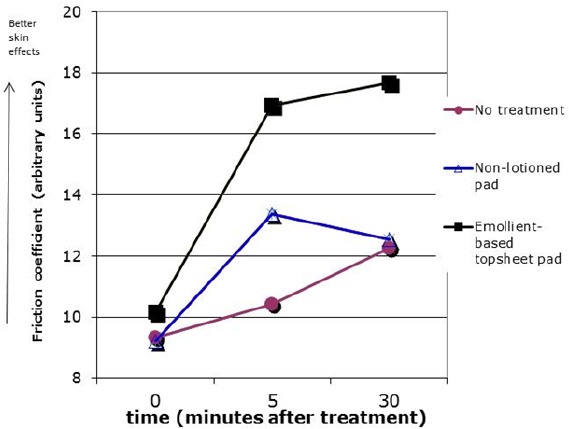
Effect of topsheet on skin friction

Skin friction study was conducted to compare two sanitary pads, one with a standard core and topsheet finish and a test pad with a foam core and emollient-based top sheet finish (Farage, Berardesca & Maibach, 2009a). Test sites were swiped 100 times with top sheets against the forearms of panelists. Friction was measure with a skin friction meter in arbitrary units prior to treatment and 5 and 30 minutes after swiping. Results (Figure 6) showed a significant increase in the skin’s coefficient of friction (change from baseline) at both time points for the test pad which is an indirect indication of a skin moisturizing effect from the lotion-containing pad (Farage, Berardesca, & Maibach, 2009a).

Coefficient of skin friction on untreated skin and skin swiped 100 times with either the test pad (foam core, emollient-based topsheet) or a reference commercial pad (a standard core and topsheet finish). Pads were swiped with the topsheet against the skin. Friction was measured with a skin friction meter in arbitrary units prior to treatment and 5 and 30 minutes after swiping. An increase in the skin’s coefficient of friction suggests an improvement in skin hydration (moisturization effect).

### 4.3 Farage Quality of Life (FQoL) Questionnaire

A proprietary Quality of Life (QoL) tool to measure the holistic impact of a consumer product on a user’s quality of life or feelings of well-being was developed and validated. The Farage Quality of Life (FQoL) tool consists of 27 general items scored on a Likert scale and covering Overall Quality of Life (1 item), Well-Being (12 items), and Energy and Vitality (14 items). The Well-Being domain has 3 subscales: Emotion, Self-Image, and Self-Competence; the Energy and Vitality domain also has 3 subscales: Personal Pleasure, Physical State, and Routine Activity (Farage, Nusair, Hansman, Sherman, & Tsevat, 2010). The FQoL tool can be completed in less than 10 minutes. To our knowledge, the FQoL was the first reproducible QoL instrument developed and validated for consumer product evaluations. It is available in 9 languages and has been used in China, Europe and South and North America.

**Figure 7:**
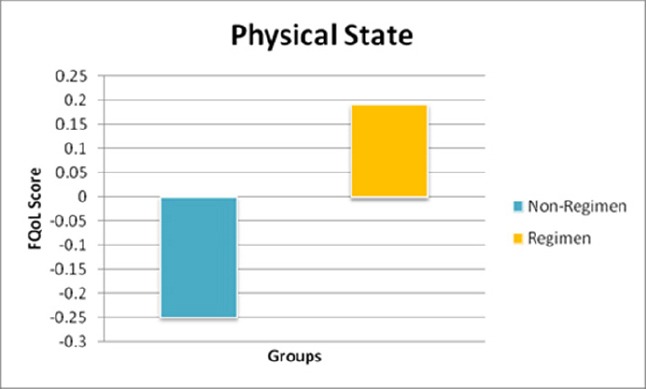
QoL Results for Panelists Using a Test Pad and Regimen over a 3-Month Period

A 3-month product usage study was conducted among 800 menstruating women. One group of 400 panelists was provided with a supply of test pads (foam core with emollient-based topsheet) to use for protection during menstruation. The other group of 400 panelists was provided a regimen of products including a supply of test pads (foam core with emollient-based topsheet) and panty liners to use as desired over the course of the 3 month study. FQoL assessments were administered at baseline, 5-7 days before the usage period began, and again 5-7 days after usage period ended. Use of the regimen of products significantly improved (p=0.0423) panelists’ physical state, indicating an improvement in well-being, over the course of the 3-month usage study (Figure 7) (Farage, Rodenberg, & Chen, 2013).

The increase in FQoL score for regimen users corresponds to an improvement in physical state which indicates an improvement in well-being.

### 4.4 Enhanced Visual Grading Using the V600^®^

As a result of the observation that unaided visual scoring can limit the ability to measure differences between very mild products, several strategies to increase method sensitivity have been explored (Farage, 2008c). Recently, cross-polarized light (V600^®^ Visualization System, Syris Scientific LCC, Gray, Maine) has been successfully used to detect the onset of subclinical irritation, i.e., changes occurring 1mm below the skin surface. Expert graders using the V600^®^ system detected significant differences between two pads in BTK testing after the first application compared to 3 applications using unaided visual scoring (Farage, 2009; Farage, Miller, Gerberick, Ryan, & Maibach, 2010; Farage, Singh, & Ledger, 2009). Recently, this approach has been patented as a clinical method. Other findings indicate that the use of the V600^®^ system has the potential to increase sensitivity of measurements across other key testing programs.

## 5. Discussion

Published reviews of clinical safety data have demonstrated that modern feminine hygiene products are effective and are not associated with clinically significant adverse dermatological, gynecological, or microbiological effects compared to conventional pads (Farage, Stadler, Elsner, & Maibach, 2004). A broader review aimed at assessing the impact of global variations in usage, skin type and ambient conditions on the skin mildness of modern sanitary pads was addressed which covered all published clinical trials conducted globally in the US, Mexico, Western Europe, Eastern Europe and Africa, These showed no significant adverse skin effects after use of modern pads compared to traditional pad designs (Farage, Elsner, & Maibach, 2007).

The urogenital health research program has yielded important insights in several areas. These include an understanding of the differences in tissue structure and physiology between the vulva and cutaneous skin; (Farage, Bjerke, Mahony, Blackburn, & Gerberick, 2003; Farage & Maibach, 2004; Farage & Maibach, 2012). Findings have also shown a correlation between visual assessments of skin irritation by expert graders and panelists’ perception of irritation (Farage, Santana, & Henley, 2005). Several approaches (Farage, 2008c) have been explored to assess differences in irritation potential between mild products including testing among panelists with sensitive skin, measurement of cytokines and the use of enhanced visual grading using cross polarized light (Farage, 2008c; Farage, Singh, & Ledger, 2009). The latter was used to reveal subsurface inflammation among patients diagnosed with vulvodynia which represents a promising new means of objectively diagnosing subclinical inflammation in disorders that have been characterized solely by sensory symptoms (Farage, Singh & Ledger, 2009; Farage, Miller, Gerberick, Ryan, & Maibach, 2010). Another research focus has been the area of sensitive skin and the impact of aging on skin. Results indicated a statistical correlation between the presence of vulvar erythema and the development of facial redness after repeated application of topical products (Farage, Bowtell, & Katsarou, 2006). A global survey of the prevalence of sensitive skin showed differences in self-reported sensitive skin with the highest reports in Asia (89.8%), followed by Europe (87.7%), Latin America (85.4%), and the US (76.8%). Overall, 68% of women claimed to have sensitive skin with 56% claiming sensitive genital skin (Farage, 2008d; Farage, Miller, Wippel, Berardesca, Misery, & Maibach, 2013). These studies were the first to establish that skin sensitivity is not limited to the facial area, but may also involve the body and the genital area. A comprehensive evaluation of skin irritation showed a consistent trend toward decreased erythema and increased irritation with age (Farage, Miller, Gerberick, Ryan, & Maibach, 2010). These findings led to recent research on the changing health and product needs of women as they age, e.g., incontinence. This research is described elsewhere (Farage, Miller, Berardesca, & Maibach, 2007; Farage, Miller, Elsner, & Maibach, 2008; Farage & Miller, 2009; Farage, Osborn, & MacLean, 2009; Farage, Miller, Berardesca, & Maibach, 2010a; Farage, 2010b; Farage, Miller, Berardesca, Maibach, & Neuhaus, 2010; Farage, Miller, & Ledger, 2010; Farage, Miller, & Maibach, 2010a; Farage, Miller, Sherman, & Tsevat, 2010; Farage, Miller, & Sobel, 2010; Farage & Miller, 2011; Farage, Miller, Ajayi, & Hutchins, 2012).

Existing data should be supplemented by larger studies, characterized by rigorous study design and statistical analysis, in which association with autoimmune diseases that show strong gender bias is explored. Further research needs include efforts to unravel the mechanisms and factors controlling neuromediators and their receptors, with the objective of finally understanding the receptor-mediated and intracellular signal pathways involved in neuroendocrine modulation of allergy and autoimmunity. For millions of women across the globe who suffer from autoimmune diseases (including cutaneous autoimmune disorders) and PMS, the ultimate research goal would be an understanding of the extremely complex interactions between the neurological, endocrine, and immune systems in order to define therapies with the potential to abort disease pathogenesis, rather than simply palliate its sequelae. PMS may represent the ideal model for these endeavors. As a disease that profoundly impacts millions of women, its etiology begs to be solved and the potential autoimmune component unraveled to facilitate appropriate and timely diagnosis as well as treatment.

Several clinical method development efforts have yielded new approaches that have been instrumental in the development of lotion-impregnated sanitary pads, tampons, wipes, facial tissues, fabrics, etc. The BTK test measures irritation under normal wear conditions and facilitates evaluation of the degree of both chemical and mechanical irritation. This method has been accepted by the FDA as a substitute for in-use clinical trials, in some cases, and approved by American Society for Testing and Materials as a global standard test method. Proprietary clinical methods were developed or adapted to enhance visual grading of irritation using cross-polarized light, to measure the amount of lotion transferred from sanitary pads and to evaluate the skin mildness of lotion-impregnated sanitary pads. Finally, the proprietary Farage Quality of Life questionnaire was used to the holistic impact of a consumer product on a user’s feelings of well-being.

## 6. Conclusion

The Feminine Care Clinical Innovation Research Program at P&G has improved women’s lives by increasing awareness of the genital health concerns of women and in helping reduce the taboo over menstruation. The research strategies described in this review paper demonstrate how a broad-based clinical innovation research program advanced the development of improved products that are transforming the lives of women world-wide, encouraged scientific knowledge and understanding in the urogenital area, and enabled the acceptance of feminine hygiene products. In addition, the research program has led to advancement of understanding of role of sex hormones in autoimmune diseases as well as aging processes that affect the physiology in multiple aspects. Specifically, this work shows how clinical safety studies can also be an effective way to advance the acceptance and use of feminine hygiene products. The efforts of the urogenital research program demonstrate how conducting and publishing basic research findings and collaborating with experts in the area have increased awareness of genital health issues and helped advance research efforts in the area. These findings have also been used to train professionals and develop materials for puberty education programs. Method development efforts have facilitated availability of tools for evaluating products’ skin benefits and have been leveraged to create products with improved comfort and genital skin health with improved quality of life for women around the world. The understanding of genital skin health along with the availability of novel measurement approaches described here will be critical in the development of new products that address the needs of women as they age.
